# The health and suffering scale: Item reduction, reliability and validity among women undergoing rehabilitation for exhaustion and long‐lasting pain

**DOI:** 10.1002/nop2.980

**Published:** 2021-07-03

**Authors:** Anja Gebhardt, Ann Langius‐Eklöf, Susanne Andermo, Maria Arman

**Affiliations:** ^1^ Karolinska Institutet Department of Neurobiology, Care Sciences and Society Stockholm Sweden; ^2^ Karolinska Institutet Department of Global Public Health Stockholm Sweden

**Keywords:** burnout, caring science, chronic pain, instrument development, measurement, nursing theory, psychometrics, rasch analysis

## Abstract

**Aim:**

To investigate the necessity of an item reduction and to evaluate estimates of dimensionality, reliability and validity of the Health and Suffering Scale among two groups of women, one undergoing rehabilitation for exhaustion and long‐lasting pain and one reference group.

**Design:**

Psychometric evaluation of the scale using cross‐sectional data.

**Method:**

The Health and Suffering Scale is a self‐report scale which measures perceived suffering in relation to health on a semantic visual analogue scale. Classical and modern test theory were applied for item reduction and to explore estimates of reliability and validity.

**Results:**

The Health and Suffering Scale was found to be unidimensional, nine of originally twenty items were part of a consistent factor structure and hierarchical order. These items were internally consistent, discriminated between patients and healthy respondents, and had an excellent level of separation of individuals experiencing various levels of health and suffering. Re‐test reliability estimates were moderate.

## 1 INTRODUCTION

Many attempts to conceptualize and measure health have been undertaken resulting in a large variety of health measurements and valuable applications in health care. Although fundamental responsibilities of nurses are to promote and restore health as well as alleviate suffering (Alligood, 2017; International Council of Nurses, 2012), existing interprofessional health measurement scales might not always be optimal to observe patients' health from a caring perspective or to evaluate acts of caring. Acts of caring relate to patients' health and suffering (Lindström et al., 2018), concepts that are complex in their definitions and are challenging to estimate numerically (Leonardi, 2018). Stakeholders in health care (Huber et al., 2016; Leonardi, 2018), including nursing theorists (Alligood, 2017) and philosophers (Gadamer, 1996; Sigurdson, 2016), emphasize the subjective, existential and dynamic nature of health. Health has been described as a movement between and integration of health and suffering or malaise (Alligood, 2017; Eriksson, 2006; Leonardi, 2018). Caring is in its roots existential (Alvsåg, 2018), thus when estimating subjectively perceived health from a caring perspective, health measurement scales also need to acknowledge the existential and dynamic relation of health and suffering.

## 2 BACKGROUND

Widely used instruments measuring health‐related quality of life are mainly conceptualized around individuals' function and symptoms as well as expectations and concerns in everyday life (Brazier et al., 1992; Herdman et al., 2011; WHO, 2002). The shortcoming of instruments developed within the medical paradigm is that existential signs of health are either rarely touched or are approached in an objectified way rather than as an inner subjective experience. We argue that nurses and caring scientists should complement each other with appropriate resources to estimate patients' health from a caring perspective. Caring science emphasizes that health cannot be understood without taking the phenomenon of suffering into consideration (Arman et al., 2015; Eriksson, 2006). Suffering is regarded to be an inseparable part of life, thus, understanding and integrating suffering into one's life is necessary in order to perceive health. Suffering that is understood and integrated into life becomes a bearable and natural part of health (Arman et al., 2015; Eriksson, 2006). According to theory, this back‐and‐forth movement between non‐integrated, unbearable suffering and integrated, bearable suffering can be affected by various coincidences in life (Eriksson, 2006).

Acknowledging these existential signs of health is specifically important in encounters with patients living with long‐term disease or patients who are going through decisive periods of life (Rehnsfeldt & Eriksson, 2004). Challenging life situations usually involve suffering and existential caring encounters are aimed at making suffering bearable through the creation of meaning (Rehnsfeldt & Eriksson, 2004). In line with an ontological understanding of health, Andermo et al. (2018) developed twenty items based on empirical data and nursing theory of health and suffering as outlined above. The items intend to capture an individual's balance of health and suffering on a visual analogue scale (VAS) between word pairs reflecting health and suffering. In the initial phase of development, the item collection was considered to be multidimensional (Andermo et al., 2018). When developing a new scale, redundant or dysfunctional items are to some degree expected but undesirable (Streiner et al., 2015). Thus, the necessity of an item reduction for psychometrical reasons must be considered in scale development. The original context of item development was in rehabilitation for long‐term disease, pain and exhaustion among mainly female patients. The reason for this was that exhaustion and long‐lasting pain are the most common reasons for sickness absence and are demanding public health problems both in Sweden (Swedish Insurance Agency, 2018, 2020) and the European Union (Breivik et al., 2013; Cimmino et al., 2011; Milczarek et al., 2009). A point of particular concern is that exhaustion and long‐lasting pain are more prevalent in women than in men (Breivik et al., 2013; Cimmino et al., 2011; Norlund et al., 2010; Purvanova & Muros, 2010; Swedish Insurance Agency, 2018, 2020). It is both a question of validity and an ethical matter that self‐report instruments used in clinical practice and research are perceived as relevant by the individuals themselves, otherwise, people's subjective perspectives risk being insufficiently considered in evidence‐based care (Hagell et al., 2009). The twenty items intend to capture individuals' inner subjective experience of their health and were found to be perceived as relevant and meaningful (Andermo et al., 2018). Maintaining continuity in the scale development, the current psychometric evaluation of the items was performed among women in a rehabilitation context. The aims of the study were to investigate the necessity of an item reduction and to evaluate estimates of dimensionality, reliability and validity of the Health and Suffering Scale among two groups of women, one undergoing rehabilitation for exhaustion and long‐lasting pain and one reference group.

## 3 DESIGN

In this second phase of development of the Health and Suffering Scale, psychometric properties of the scale and the items were tested using cross‐sectional data.

## 4 METHOD

### 4.1 Sample

Women were selected consecutively at a rehabilitation clinic for exhaustion and long‐lasting pain in Sweden. There were 297 eligible patients and the response rate after two reminders was 56.2%, yielding 167 participants. One participant's responses in the Health and Suffering Scale (HSS) were all missing and were excluded from the analysis, yielding 166 participants. The sample consisted of 94 participants (56.6%) undergoing rehabilitation for long‐lasting pain and 69 participants (41.6%) undergoing rehabilitation for exhaustion. Three participants (1.8%) received another kind of rehabilitation or had finished the rehabilitation program more than 3 months before (Table 1). More than a third of women (39.8%, *N* = 66) were working in human service professions, including childcare and teaching.

**TABLE 1:** Sociodemographic characteristics of participants

	Patients	Students
*N* = 295	*N* = 166	*N* = 129
	*N* (%)	*N* (%)
Long‐term sick leave	100 (59.9%)	0 (0%)
Rehabilitation		
Long‐lasting pain	94 (56.6%)	8 (6.2%)
Exhaustion	69 (41.6%)	0
Other/no rehabilitation	3 (1.8%)	121 (93.8%)
Sociodemographic variables		
Age, years, mean ± Std	47.7 ± 11.1	38.1 ± 7.7
Marital status		
Single	58 (34.9%)	21 (16.3%)
Married/cohabiting	108 (65.1%)	108 (83.7%)
Family		
Mothers	129 (77.7%)	108 (83.7%)
Education		
Comprehensive school	11 (6.6%)	0
Secondary School	59 (35.5%)	0
Higher education/University	96 (57.8%)	129 (100%)
Employment status		
Employed	73 (44.0%)	115 (89.1%)
Self‐employed	11 (6.6%)	2 (1.6%)
Student	5 (3.0%)	123 (95.3%)
Retired	14 (8.4%)	1 (0.8%)
Off‐duty/parental leave	2 (1.2%)	21 (16.3%)
Job seeking	12 (7.2%)	0 (0%)
Home worker	7 (4.2%)	5 (3.9%)
Other/Nothing	18 (10.8%)	2 (1.6%)

A reference sample consisted of nurses, occupational therapists and physiotherapists studying in health care programs for specialization within their profession at a medical university, Sweden. The sample was selected consecutively for known‐group validation and calculation of test re‐test reliability. Students on specialization level were chosen for known‐group validation foremost because they were expected to be healthy women which is a central criterion for testing the scale's ability to differentiate between healthy and suffering women. Further, they matched to some degree patients' employment within human service profession and midlife situation. There were 209 eligible students and the response rate after two reminders was 61.7%, yielding a final sample of 129 participants. Response rate for the re‐test was 83.7% (*N* = 108). Eight students (6.2%) reported having received rehabilitation for long‐lasting pain or exhaustion during the last 3 months.

### 4.2 Instruments

**FIGURE 1:**
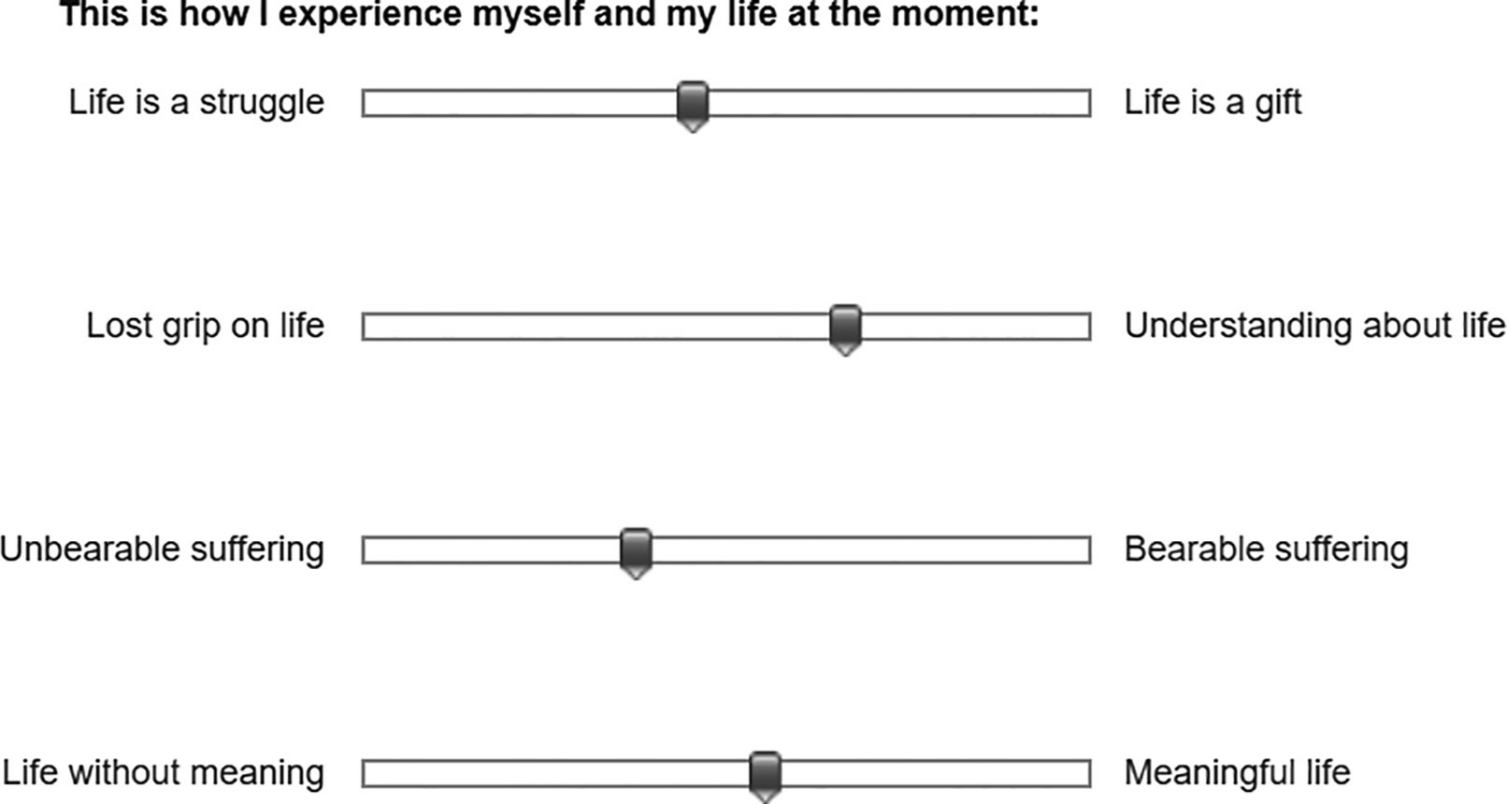
Example of four Health and suffering scale items taken from the web survey

The Health and Suffering Scale (HSS) was developed on both an empirical and theoretical basis (Andermo et al., 2018). It is a self‐report scale consisting of 20 items that intends to measure perceived suffering in relation to health on a semantic visual analogue scale (VAS). Perceived suffering is reported in relation to perceived health on a VAS between word pairs reflecting health and suffering according to the theory of Eriksson (2006), for example, “lost grip on life – understanding about life” or “life without meaning – meaningful life” (Figure 1). Two of the 20 items directly reflect the concepts of health and suffering: “Barriers to health – Health” and “Unbearable suffering – bearable suffering”. The remaining 18 items were initially related to five sub‐domains of health and suffering: life passion and energy, presence in life, relationships, personal freedom and meaning. The VAS registers 101 steps from 0 to 100. A low score on an item reflects perceived unbearable suffering and health hindrances in life whereas a high score on an item reflects bearable suffering and the experience of health in life. None of the items was reverse scored.

The Karolinska Exhaustion Disorder Scale (KEDS) is an established clinical measure of exhaustion (Beser et al., 2014) and was used for known‐group and convergent validation. KEDS is a self‐report scale consisting of nine items measuring the degree of stress‐induced exhaustion by estimating ability to concentrate, memory, physical stamina, mental stamina, recovery, sleep, sensory impressions, experience of demands and irritation and anger. Each item is estimated on a seven‐point Likert scale. A high sum score reflects a high degree of exhaustion. In the current samples, the internal consistency of the KEDS was α = 0.84 (CI 0.81–0.87) for patients and α = 0.88 (CI 0.85–0.9) for healthy individuals.

Sociodemographic data were obtained using item formulations suggested by the government agency *Statistics Sweden* (SCB, 2004).

### 4.3 Data collection

Patients and students were orally informed about the study in the middle of the 10‐weeks rehabilitation period and in connection to a lecture, respectively, and could voluntarily sign up for an information letter including a personal link to the study's web survey by email. A first and second reminder was sent out after 2 and 4 weeks after the information letter had been sent. Data were collected between October 2018 and August 2019.

Participants entered the web survey by a personal link. The web survey was identical for both samples, consisting of sociodemographic questions, HSS, KEDS and a third instrument not evaluated in the current study. Patients were asked to complete the survey once, whereas students were asked to complete the HSS for a re‐test 2 weeks after having submitted the survey for the first time. Students were asked to participate in a re‐test because they were expected to be more stable in their health experience than patients under rehabilitation. The median (IQR) time between answering test and re‐test was 15 (13–20) days.

## 5 ANALYSIS

Psychometric hypotheses guiding the analysis:

1. Dimensionality

a. The HSS is expected to be multidimensional with factors having a reasonable share of the total variance.

b. The factor structure is simple (items load only on one factor) and consistent between both samples.

2. Item reduction

a. Item reduction will be necessary for items with a unique variance >0.5 and inconsistent hierarchical position between samples.

3. Reliability estimation

a. Coefficient α is between 0.7 and 0.9 for each extracted factor.

b. The paired differences of the test and re‐test sample come from a distribution with zero median and the ICC estimate is categorized as substantial (> 0.8).

c. The HSS can separate between the two samples and individuals perceiving various levels of suffering within each sample.

4. Construct Validation

a. Item hierarchy is meaningful from a theoretical point of view and consistent in both samples.

b. The HSS targets the patient group better than the reference sample and differentiates between the two samples.

c. Correlations between the constructs of HSS and KEDS are moderate (0.4 – 0.69) (Schober et al., 2018).

### 5.1 Dimensionality and item reduction

Factor analysis was applied separately in the two data sets, both to explore dimensionality of HSS and for item reduction (MatLab version R2019b, MathWorks). Factor solutions consisting of factors with eigenvalues >1 were investigated and estimated factor loadings were rotated according to both the varimax and promax method. The communality of a variable should be >0.5 and a factor should not consist of fewer than three variables (Norman & Streiner, 2014). Factor loadings exceeding 0.5 are considered practically significant and factor loadings >0.7 indicate a clear factor structure (Hair et al., 2016).

The MatLab function ‘factoran’ returns maximum likelihood estimates of the factor loading matrix and the specific variances. The patient sample skewness was −0.14 and kurtosis was 2.36. The distribution of the reference sample was moderately skewed to the left (−0.69) and had a kurtosis of 3.36. Thus, the assumptions of normality were not severely violated considering that a normal distribution has a skewness of 0 and a kurtosis of three. Further, exploratory factor analysis is robust against moderate deviations from normality (Norman & Streiner, 2014).

### 5.2 Reliability estimation and construct validation

The Andrich rating‐scale model (RSM) within Rasch analysis was applied (Winsteps version 4.5.3, Winsteps & Facets Rasch Software) to refine item reduction and explore construct validity, rating scale properties, item function and ability to separate between individuals perceiving various levels of suffering. Prior to analysis, the 101‐step VAS was transformed to five response categories. A category is regarded to function reasonably well when it has a distinct probability peak (>0.5) for a certain part of the measured variable, when the distance between threshold estimates are >1.4 logits and <5 logits and when the outfit mean square (MnSq) <2 when evaluating category fit (Bond & Fox, 2015). A reasonable MnSq range for item fit in rating scales is 0.6 < 1.4. A low MnSq might indicate redundant item responses (Wright & Linacre, 1994). An instrument discriminates a sample into three or four levels for person reliability >0.9 and a person separation index of three has been described as an excellent level of separation (Boone et al., 2013).

Internal consistency was evaluated with Cronbach's alpha coefficient (Leontitsis, 2020). Intraclass coefficient (ICC) was calculated to estimate test re‐test reliability according to Shrout and Fleiss (1979), convention (2,1), with a 95% confident interval (Qin et al., 2019; Zoeller, 2020). The ICC was selected based on the following assumption: In the test re‐test, subjects provided observations for both measurement occasions which require an analysis by a 2‐way model (Weir, 2005). Addressing both systematic and random error in the estimation of the ICC was of interest in order to take agreement (versus consistency) of individual observations between the two measurements into account (McGraw & Wong, 1996; Weir, 2005). McGrow & Wong's absolute‐agreement, 2‐way mixed‐effects model (A,1) corresponds to Shrout and Fleiss (1979) model (2,1) (Weir, 2005). Shrout (1998) proposed categorizing ICC estimates 0–0.1; 0.11–0.4; 0.41–0.6; 0.61–0.8 and 0.8–1.0 as none, slight, fair, moderate and substantial, respectively.

Wright maps and construct key maps according to the RSM were used to compare visually between the samples to assess item hierarchy and reliability of the construct. A well‐targeted instrument has a mean person estimate that is close to the mean item difficulty (Bond & Fox, 2015).

Moreover, HSS construct was evaluated correlating the individual sum scores of the new nine‐item‐scale (reduced to five response categories) to the sum scores of KEDS with the Pearson coefficient. Unpaired and paired sample *t* test were used to compare distributions of the patient and student sample. Effect sizes for parametric tests were calculated using Cohen's *d* (Cohen, 1988, as cited in Fritz et al., 2012) with values >0.8 implying a large clinical significance. Assumptions of normal distributions were not severely violated.

Health and Suffering Scale data were missing for 9.8% of observations among patients versus 1.6% among students. In all the missing values in the patient sample, no slider movement was registered. The VAS slider in the web survey was on zero by default and participants might not have moved the slider because it already appeared to be in the position zero, reflecting unbearable suffering. In comparison, the percentage of missing values in KEDS‐generated data was 1.4% among patients versus 0% among students. We assumed the percentage of missing values in the HSS‐generated data to be equally distributed as observed in the student data and the KEDS‐generated data. Missing values were therefore treated as value zero.

## 6 ETHICS

Ethical approval for this study was obtained from the Swedish Ethical Review Authority (nr 2015/4:3, nr 2016/993‐32 and nr 2018/1681‐32). Participants received written information that submitting the survey implied giving consent for study participation.

## 7 RESULTS

Women in the patient sample were older, had a lower education, were more often single, and were more often job seeking than women in the reference sample (Table 1). Women in the patient sample reported a significantly higher degree of exhaustion (Mean = 41.1; Std = 8.0) than women in the reference sample (Mean = 25.8; Std = 8.7) as measured by the KEDS; t (293) = 15.72, CI 13.42–17.26, *p* < .001, *d* = 1.85.

### 7.1 Dimensionality

The exploratory factor analysis of the patient data identified two factors with eigenvalues >1 (11.2, accounting for 56% of the total variance and 1.4, accounting for 7% of the variance of the 20 items). In the reference sample, three factors with eigenvalues >1 were identified (9.7, accounting for 48.5% of the total variance, 2.1 accounting for 10.5% of the total variance and 1.2, accounting for 6% of the total variance of the 20 items). Biplots and deep scree plots indicated a strong first factor.

After this preliminary factor extraction, the ‘varimax’ rotation distributed the variance most equally for the 2‐factor solution in the patient sample resulting in eigenvalues of 7.7 and 4.2 for the two factors. The ‘promax’ rotation provided the most equal distribution for the 3‐factor solution in the reference sample with eigenvalues of 3.7, 3.5 and 3.3 for the three factors. This more equitable division after rotation was not reflected in the distribution of the item loadings revealing a complex factor structure with most variables still loading strongly on the first factor in both samples. The varimax solution of the factor structures between the samples for item loadings of >0.5 revealed that nine items were identical in the first factor and that 4 items were identical in the second factor. The nine identical items in the first factor had a unique variance of <0.5, whereas the four identical items in the second factor had a unique variance of >0.5. The comparison indicated inconsistency in the 2‐factor solution between the two samples and HSS was explored for unidimensionality.

The 1‐factor solutions showed that all items loaded >0.5 in the patient sample compared to13 items in the reference sample, implying that more than 25% of the variance of most items can be explained by one factor (Hair et al., 2016) (Table 2). Analysing both samples as one sample (*N* = 295) revealed a strong first factor with an eigenvalue of 12.16 (explaining 60.8% of the total variance) and a second factor with an eigenvalue of 1.46 (7.3% of the total variance).

**TABLE 2:** Factor loadings and unique variances for the 1‐factor solution in patient sample and reference sample

Item	Patient sample (*N* = 166)		Reference sample (*N* = 129)	
	Factor loading	Unique variance	Factor loading	Unique variance
Resigned – Faith and hope in future	0.873	0.237^a^	0.864	0.254^a^
Life is dark – Life is bright	0.869	0.244^a^	0.826	0.318^a^
Life without value – Valuable life	0.863	0.256^a^	0.856	0.267^a^
Lost grip on life – Understanding about life	0.847	0.280^a^	0.853	0.273^a^
Life without meaning – Meaningful life	0.844	0.287^a^	0.888	0.212^a^
Tired of life – Life passion	0.817	0.333^a^	0.893	0.203^a^
Tired of struggling – Engagement in life	0.805	0.352^a^	0.809	0.346^a^
Unbearable suffering – Bearable suffering	0.794	0.370^a^	0.764	0.416^a^
Life is a struggle – Life is a gift	0.763	0.417^a^	0.771	0.406^a^
Stuck in negative life patterns – In a process of development	0.753	0.434^a^	0.637	0.594
Worried about the future – Feeling safe in relation to future	0.735	0.460^a^	0.672	0.549
Chaos – Order	0.667	0.555	0.431	0.814
Abstracted – Present	0.665	0.558	0.521	0.728
Lonely struggle – Supporting communion	0.634	0.598	0.472	0.777
Pressured by demands – Relaxed	0.625	0.610	0.352	0.876
Run over myself – Listen to myself	0.608	0.630	0.410	0.839
Unsatisfactory relations – Loving relations	0.600	0.639	0.458	0.791
Have a shelter or wall around myself – Can be myself	0.585	0.658	0.491	0.759
Barriers to health – Health	0.564	0.682	0.559	0.688
Lack of energy – Filled with energy	0.519	0.730	0.478	0.771

^a^ Unique item variance <0.5.

### 7.2 Item reduction

Following a 1‐factor solution, items with a unique variance of >0.5 were excluded (Table 2). According to this criterion, nine items were suggested to be retained with total consistency between the samples: ‘Life is a gift’, ‘Valuable life’, ‘Meaningful life’, ‘Life is bright’, ‘Life passion’, ‘Faith and hope in future’, ‘Engagement in life’, ‘Understanding about life’, and ‘Bearable suffering’. Additionally, two items had a unique variance of <0.5 in the patient sample: ‘In a process of development’ and ‘Feeling safe in relation to future’. The latter item seemed to be redundant with the item ‘Faith and hope in future’ and was excluded whereas the item ‘In a process of development’ was retained because it was regarded to be important from a theoretical point of view. The 1‐factor model with 10 items retained explained 68.3% of the total variance in the patient sample and 66.7% in the reference sample. All 10 retained items loaded >0.7 in both samples (with the exception of the item ‘In a process of development’ in the reference sample >0.5) implying that >50% of the variance of the variables was accounted for by one factor.

Applying RSM for comparison of item hierarchies between the two samples and the re‐test reference sample (Table 3) revealed a reasonably consistent structure of item difficulty. The structure showed that items clustered into three hierarchical groups consisting of three variables each, with one exception; the item ‘Tired of life – Life passion’ changed its hierarchical position dependent on the samples and it was decided to be excluded.

**TABLE 3:**
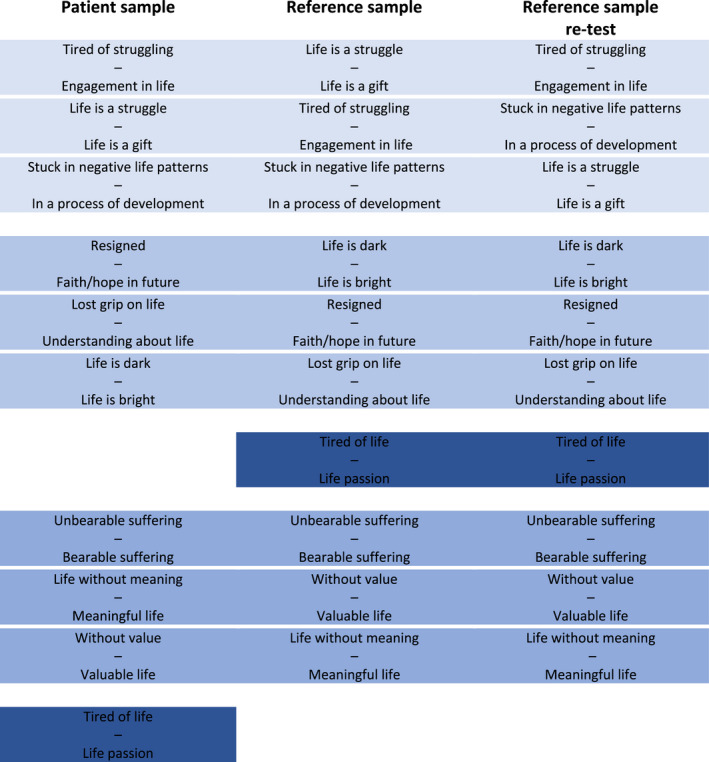
Item difficulty of the health and suffering scale

Note

Ordered from the most difficult items at the top to the easiest item at the bottom.

### 7.3 Reliability estimation

The probability curves of categories within the RSM showed that each part of an item measure was assigned one most probable response category with distinct probability peaks >0.5. The distances between threshold estimates ranged from 1.64 to 2.39 for the patient sample and from 1.5 to 2.56 for the reference sample, category outfit MnSq were <2 and almost all close to 1, suggesting functional category fit for the chosen reduction from 101 to five categories.

The MnSq outfit for item reliability ranged from 0.71 to 1.35 in the patient sample and from 0.57 to 1.71 in the reference sample, indicating that the two items ‘Bearable suffering’ (MnSq = 1.5; Zstd 2.39) and ‘In a process of development’ (MnSq = 1.71; Zstd 4.41) were underfitting the RSM in the reference data set (higher share of unpredicted responses in relation to respondents' ability). Real person reliability for non‐extreme (*N* = 161) persons out of the patient sample was 0.92 and 0.87 for non‐extreme (*N* = 118) persons out of the reference sample item. Real person separation was 3.29 for the patient sample and 2.57 for the reference sample indicating that HSS has a good to excellent level of separation. The difference of the HSS sum scores between the two samples was significant; t (293) = 11.89, 95% CI 9.0–12.56, *p* < .001; *d* = 1.4 (see known‐group comparison, Table 4).

**TABLE 4:** Health and suffering scale mean sum score values for known‐group comparison and test‐retest comparison

	Known‐group comparison		Test‐retest comparison	
	Patient sample	Reference sample	Reference sample test	Reference sample retest
	*N* = 166	*N* = 129	*N* = 108	*N* = 108
Mean (Std)	26.4 (8.4)	37.2 (6.8)	29.3 (6.2)	29.0 (6.1)
*p*‐value	<.001^a^		>.05^b^	
Cohen's *d*	1.4^c^		0.05^c^	

^a^ Unpaired sample *t* test.

^b^ Paired sample test.

^c^ Effect size estimate for parametric tests according to Fritz et al. (2012).

Internal consistency for the nine retained variables was overly high in both the patient sample (α = 0.95; CI 0.94–0.96) and the reference sample (α = 0.94; CI 0.93–0.95), indicating item redundancy. ICC_2,1_ estimates (reference sample only) for the nine items ranged from 0.42 (‘Bearable suffering’) to 0.64 (‘Meaningful life’). The ICC_2,1_ estimate for the sum of all nine‐item observations was 0.63 (CI 0.5–0.73), indicating fair to moderate ICC_2,1_ estimates of the nine retained items. The paired differences of the test and re‐test sample were not significant; t (107) = 0.6, CI −0.71–1.32; *p* > .05 (see test‐retest comparison, Table 4).

### 7.4 Construct validation

**FIGURE 2:**
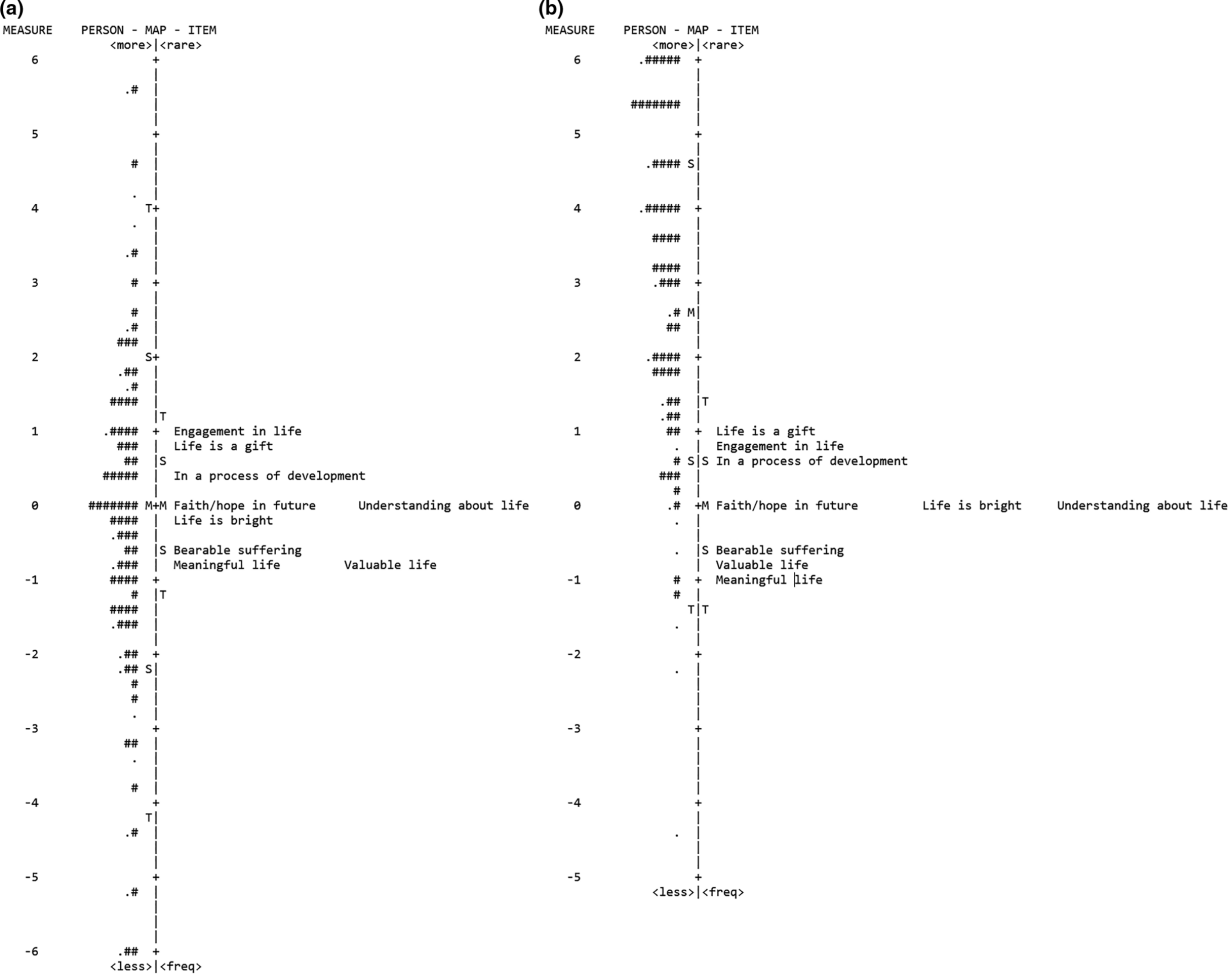
(a) Wright map of the patient sample. Items are ordered on the right side from the most difficult items at the top to the easiest item at the bottom. Persons are ordered on the left side from individuals with the highest perceived health at the top to individuals with the lowest perceived health at the bottom. (b) Wright map of the student sample. Items are ordered on the right side from the most difficult items at the top to the easiest item at the bottom. Persons are ordered on the left side from individuals with the highest perceived health at the top to individuals with the lowest perceived health at the bottom

The Wright map of the patient sample (Figure 2a) revealed the mean person estimate (−0.26 logits) to be close to the mean item difficulty implying that the difficulty of the HSS items matched the ability of the patients well. Item difficulty was distributed narrowly between two standard deviations (∼ −1.2 logit <1.2 logit) from the mean item measure, whereas patients' ability was distributed widely between two standard deviations (∼ −4.1 logits <4 logits) from the mean person measure. Thus, the HSS might not sufficiently separate patients' perceived suffering outside the narrow ability range of the existing items. The Wright map of the reference sample (Figure 2b) revealed the mean person estimate to be higher (2.88 logits) than the mean item difficulty illustrating that the HSS does not target the ability of the reference sample. The item difficulty was almost similarly distributed as in the patient data set. In contrast, students' ability was distributed top heavy (∼ −1 logit <6.8 logits) indicating that it is relatively easy for the students to rate high on the scale's items (implies low perceived suffering/high perceived health). According to the maps, the threshold to rate high on a ‘Meaningful life’ and ‘Bearable suffering’ is low in relation to the own constitution (ability), in contrast, the threshold to rate high on ‘In a process of development’ and ‘Engagement in life’ is high in relation to the own constitution and the other items. From a theoretical perspective, the hierarchical structure was assessed to be meaningful.

The scores on the HSS correlated negatively with the scores on the KEDS in both the patient sample (*rho*_P_ = −0.58; CI −0.82 – −0.51) and the reference sample (*rho*_P_ = −0.42; CI −0.63 – −0.28). Thus, the less perceived health and the more suffering in life was reported, the higher degree of exhaustion.

## 8 DISCUSSION

The current study found the HSS to be of unidimensional character; nine of originally twenty items were part of a consistent factor structure and hierarchical order in two different samples of women. These items were internally consistent and discriminated between patients and healthy respondents. Further, they showed to have an excellent level of separation of individuals experiencing various levels of suffering in each sample. However, re‐test reliability in a reference sample was estimated to be fair to moderate among a sample of women who were expected to be more stable regarding their perceived health and suffering.

The study's major finding is that the original, supposedly multidimensional, instrument turned out to be unidimensional and that only nine of 20 items were retained, a procedure that risks changing the scale's core construct and therefore critically needs to be discussed. Those nine items which contributed to a consistent factor structure in two different but also comparable contexts were closely related to caring science theory on health and suffering. From previous research and philosophical concepts of health and suffering, it is already known that engagement in life and developmental processes are signs of health, in contrast to suffering as a state of paralysis or struggle of the self in life (Bueno‐Gómez, 2017; Eriksson, 2006; Morse, 2001; Rehnsfeldt & Eriksson, 2004; Svenaeus, 2015; Wiercinski, 2013). The importance of an understanding about life for the own health has previously been emphasized by Rehnsfeldt and Eriksson (2004) who described lack of life understanding as an experience of darkness and unbearable suffering. Finally, the experience of meaning in life was found to be a strong underlying factor in the evaluation of the scale's construct. Thus, an important aspect that needs to be discussed is whether the item reduction changed the scale's intended measure of perceived health and suffering to a measurement of perceived meaning in life. Discussing this aspect requires a closer look at the content of items excluded from the scale. Excluded items mainly related to relationship to others or oneself (e.g. “Lonely struggle – supporting communion”; “Have a shelter around myself – can be myself”) and perception of life (e.g. “Chaos – order”; “Pressured by demands – relaxed”). Regarding content, the excluded items were related to health and suffering, but may not be as central to the concept as the retained items, which was supported empirically by the statistical analysis. In conclusion, the content validity of the scale as a measure of health and suffering was not diminished by the item reduction; rather, it might have become more rigorous. For the measurement itself as well as for theory, it is important to take notice of the strong statistical position of perceived meaning in life within the construct of health and suffering. This phenomenon might empirically emphasize that life meaning is inseparably bound up with perceived health and suffering. As a consequence, health and suffering can neither be fully understood nor defined without taking the subjective existential meaning of ailments or health hindrances for the individual life into account (Bueno‐Gómez, 2017; Sigurdson, 2016; Svenaeus, 2015). Thus, exploration of the scale's dimensions and item reduction in combination with the Rasch analysis yielded important empirical knowledge about the theory of health and suffering.

Reliability coefficients reflect the amount of error and “the extent to which a measurement instrument can differentiate among individuals” (Streiner et al., 2015, p. 161). The ICC estimates indicated a moderate reliability of test scores among healthy women, whereas the Rasch analysis revealed an excellent level of separation indicating that the HSS has the prerequisites to separate individuals into three to four groups of various perceived suffering. One reason explaining these slightly different results might be that a low ICC estimate can be a sign of low between‐subject variability in the sample (Streiner et al., 2015; Weir, 2005). Signs of low between‐subject variability in the reference sample could be found in the Wright map. The Wright map of the reference sample illustrated that most individual scores were high indicating ceiling effects with the risk to not sufficiently separate students' perceived suffering outside the narrow ability range of the existing items. The scale targeted the patient sample much better, as illustrated by the corresponding Wright map, which leads to the conclusion that higher between‐subject variability might be expected and consequently higher estimates of reliability in the patient sample. One limitation of the current study is that a test re‐test was not performed in the patient sample because the patients were not regarded to be stable in their health and suffering experience when undergoing rehabilitation. Even though reliability estimates are rather bound to the interaction between a specific study context and the instrument than the instrument itself (Shrout, 1998; Streiner et al., 2015), the obtained ICC estimates have implications for the power calculation of studies using the scale. In future studies, it has to be taken into account that sample size needs to be adjusted to the probability of detecting a true effect which in turn depends on the ability of the HSS to differentiate among individuals, its reliability (Shrout, 1998; Streiner et al., 2015; Weir, 2005).

For convergent validation of the HSS, a validated clinical measure was needed that captured the health condition of both patients with pain and those with exhaustion. As pain disorders and symptoms of exhaustion disorder tend to co‐occur (Borchers & Gershwin, 2015; Eller‐Smith et al., 2018; Salvagioni et al., 2017; Yalcin & Barrot, 2014), the assessment instrument for exhaustion disorder syndrome, KEDS (Beser et al., 2014), was chosen as a clinical convergent measure. When evaluating convergent validity, the association between the new construct and a similar established construct should be robust, but not “overly high” (Streiner et al., 2015, p. 240). A moderate association between exhaustion and perceived health and suffering was found, and according to the confidence interval it can be considered robust at least for the patient sample. This indicates that estimates of exhaustion are specific to the clinical context but not equivalent to the construct of health and suffering which is more general and not bound to a specific clinical context. Although pain and symptoms of exhaustion co‐occur (Yalcin & Barrot, 2014), the KEDS is no absolute measure for the health of the heterogenic patient group of patients suffering from pain and/or exhaustion. This effect might have contributed to a moderate association between clinically determined health and perceived health and suffering.

### 8.1 Limitations

The psychometric properties of the HSS were evaluated within patients undergoing rehabilitation for long‐lasting pain and exhaustion as well as in students in health care programs, contexts that are strongly dominated by female individuals. Thus, further evaluation of the HSS in male populations is needed. A major drawback of the chosen reference sample is that respondents were not selected from the Swedish normal population but from a university, causing significant differences in the age and educational level between the samples. Further, the requirement of a sufficient sample size for factor analysis was only fulfilled at a minimum level with subject to item ratios of 8:1 and 6:1. The statistical risks are that the items loaded on the wrong factor with a misleading factor structure as a consequence (Norman & Streiner, 2014). However, analysing both samples as one, implying a subject to item ratio of almost 15:1, reinforced the unidimensional structure of the scale. According to the item separation indexes obtained in both samples (>4, item reliability >0.9), the sample size needed for a reliable Rasch analysis was fulfilled (Boone et al., 2013). Another major limitation is the high percentage of missing items in the HSS due to the deceptive appearance of the digital slider function.

## 9 CONCLUSION

**FIGURE 3:**
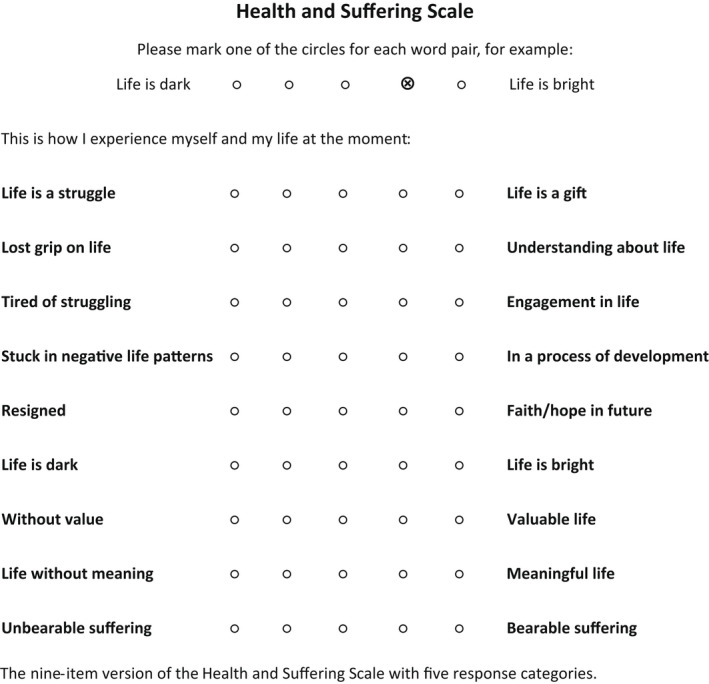
The nine‐item version of the Health and suffering scale with five response categories

The study identified a HSS nine‐item version including five response categories (Figure 3) to be a unidimensional measure of perceived health and suffering with reasonable estimates of reliability and validity among women undergoing rehabilitation for exhaustion and pain. The scale reflects an ontological understanding of health as subjective, existential and dynamic, embracing suffering as a natural part of life. After further psychometric investigation, the HSS is expected to help patients and health care professionals guide and evaluate rehabilitation processes that aim to enhance individuals' health and alleviate their suffering. This psychometric evaluation of the HSS serves as a first basis for future studies with the aim to estimate patients' subjective health and suffering from a caring perspective within the context of rehabilitation of exhaustion and long‐lasting pain. Nevertheless, we advise, in line with general recommendations of psychometricians, to re‐assess psychometric properties of the HSS in parallel with future investigations' main aim.

## CONFLICT OF INTEREST

The authors have no conflicts of interest to declare. All co‐authors have seen and agree with the contents of the manuscript and there is no financial interest to report. We certify that the submission is original work and is not under review at any other publication.

## AUTHOR CONTRIBUTIONS

AG, SA, and MA designed the study. AG collected the data, performed the analysis, and wrote the manuscript. The analysis was scrutinized by AL and critically discussed by all authors. All authors contributed to the final manuscript and approved to submission.

## Data Availability

Raw data are archived at Karolinska Institutet, Sweden. Data supporting the findings of this study are available from the corresponding author AG on reasonable request.
